# Ventricular-Vascular Uncoupling in Heart Failure: Effects of Arterial Baroreflex-Induced Sympathoexcitation at Rest and During Exercise

**DOI:** 10.3389/fphys.2022.835951

**Published:** 2022-04-05

**Authors:** Joseph Mannozzi, Mohamed-Hussein Al-Hassan, Jasdeep Kaur, Beruk Lessanework, Alberto Alvarez, Louis Massoud, Tauheed Bhatti, Donal S. O’Leary

**Affiliations:** ^1^ Department of Physiology, Wayne State University School of Medicine, Detroit, MI, United States; ^2^ Department of Kinesiology and Health Education, University of Texas at Austin, Austin, TX, United States

**Keywords:** arterial baroreflex, ventricular-vascular coupling (VVC), baroreceptor unloading, orthostatic hypotension, orthostatic intolerance

## Abstract

Autonomic alterations in blood pressure are primarily a result of arterial baroreflex modulation of systemic vascular resistance and cardiac output on a beat-by-beat basis. The combined central and peripheral control by the baroreflex likely acts to maintain efficient energy transfer from the heart to the systemic vasculature; termed ventricular-vascular coupling. This level of control is maintained whether at rest or during exercise in healthy subjects. During heart failure, the ventricular-vascular relationship is uncoupled and baroreflex dysfunction is apparent. We investigated if baroreflex dysfunction in heart failure exacerbated ventricular-vascular uncoupling at rest, and during exercise in response to baroreceptor unloading by performing bilateral carotid occlusions in chronically instrumented conscious canines. We observed in healthy subjects that baroreceptor unloading caused significant increases in effective arterial elastance (Ea) at rest (1.2 ± 0.3 mmHg/ml) and during exercise (1.3 ± 0.2 mmHg/ml) that coincided with significant increases in stroke work (SW) (1.5 ± 0.2 mmHg/ml) and (1.6 ± 0.2 mmHg/ml) suggesting maintained ventricular-vascular coupling. Heart Failure significantly increased the effect of baroreceptor unloading on Ea at rest (3.1 ± 0.7 mmHg/ml) and during exercise (2.3 ± 0.5 mmHg/ml) whereas no significant increases in stroke work occurred, thus signifying further ventricular-vascular uncoupling. We believe that the enhanced ventricular-vascular uncoupling observed during baroreceptor unloading only worsens the already challenged orthostatic and exercise tolerance and thereby contributes to poor exercise performance and quality of life for heart failure patients.

## Introduction

Beat by beat control of blood pressure is crucial to the maintenance of cardiovascular performance. The primary mechanism by which blood pressure control occurs is through arterial baroreflex mediated changes in sympathetic and parasympathetic activity to modify heart rate and vascular resistance which thereby maintains blood pressure within normal ranges in response to changes in posture, exercise, and activation of other cardiovascular reflexes or local vascular responses (Melcher and Donald, 1981; Chen et al., 1991; Olivier and Stephenson, 1993; Iellamo et al., 1997; DiCarlo and Bishop, 2001; Joyner, 2006; Sheriff, 2006; Drew et al., 2008; Chen et al., 2010; Davis, 2011; Fadel, 2015).

In heart failure arterial baroreflex regulation is significantly altered. Most notably the ability of the baroreflex to increase blood pressure in response to a hypotensive stimulus is attenuated and this is primarily an effect of an attenuated ability to increase cardiac output due to depressed ventricular function (Chen et al., 1991; Dall'Ago et al., 1997; DiCarlo and Bishop, 2001; Kim et al., 2004; Kim et al., 2005a; Olivier and Stephenson, 1993; Osterziel et al., 1995; Thames et al., 1993; Wang et al., 1996). Furthermore, the range and gain of heart rate responses to baroreceptor unloading is significantly depressed whereas the overall change in vasoconstriction remains largely unaffected (Olivier and Stephenson, 1993; Osterziel et al., 1995; Kim et al., 2004; Joyner, 2006). Sympatho-inhibitory responses and spontaneous baroreflex control are also significantly attenuated engendering sympatho-excitatory responses with attenuated negative feedback control (Ferguson et al., 1992; Drew et al., 2008; Ichinose et al., 2008; Chen et al., 2010). Thus, alterations in baroreflex function are variable under cardiovascular pathologies and require an assessment of the output effects of the respective increases or decreases in baroreflex function and how they relate to overall cardiovascular efficiency. One such measure is an assessment of ventricular-vascular coupling that can be assessed by changes in arterial elastance and stroke work (Sunagawa et al., 1985; Mannozzi et al., 2020).

Optimal ventricular-vascular coupling is paramount to the maintenance of efficient cardiovascular performance at rest and during exercise (Sunagawa et al., 1983; Sunagawa et al., 1985; Burkhoff and Sagawa, 1986; Nozawa et al., 1988; Asanoi et al., 1989; Kelly et al., 1992a; Kelly et al., 1992b; Eichhorn et al., 1992; Hayashida et al., 1992; Little and Cheng, 1993; Kass, 2005; Chantler et al., 2008; Otsuki et al., 2008; Cote et al., 2013; Ky et al., 2013; Mannozzi et al., 2020). This relationship is a ratio of Effective Arterial Elastance (E_a_) vs. ventricular maximal elastance (E_MAX_). E_a_ is a vascular property that was originally derived by relating total peripheral resistance with systolic, and diastolic time periods, as well as the diastolic time decay constant to assess the load imposed by the vasculature on the left ventricle (Sunagawa et al., 1983; Sunagawa et al., 1985; Kelly et al., 1992a). Later this series of variables was simplified by Kelly et al. (1992a) for assessments of vascular load in humans to the relationship of end systolic pressure divided by stroke volume. Within that series of derivations another method for measuring E_a_ focusing on vascular parameters utilizes total vascular resistance divided by cardiac cycle (reference). This latter method showed limited bias and statistical difference from the fully derived method (Kelly et al., 1992a; Mannozzi et al., 2020). Either method is valid for assessing E_a_ and they have been used to define the state of ventricular-vascular across a wide range of settings and pathologies (Sunagawa et al., 1985; Asanoi et al., 1989; Little and Cheng, 1991; Kelly et al., 1992a; Redfield et al., 2005; Otsuki et al., 2006; Chantler et al., 2008; Otsuki et al., 2008; Cote et al., 2013; Faconti et al., 2017; Reddy et al., 2017; Mannozzi et al., 2020). To date no study has quantified the effect of baroreflex activation on ventricular-vascular coupling nor on the impact of heart failure on baroreflex mediated changes in ventricular-vascular coupling at rest and exercise. In this study we hypothesized that in normal subjects arterial baroreflex unloading at rest and during exercise would cause improved ventricular-vascular coupling to produce optimal blood flow and thereby likely allow for timely, appropriate corrections in blood pressure. In heart failure the alterations in baroreflex control of arterial elastance will lead to further worsening of the already uncoupled ventricular-vascular relationship due to an inability to improve stroke work in parallel with increases in E_a_, and this likely contributes to orthostatic and exercise intolerance.

## Methods

Five (1 male 4 females) adult mongrel canines 18–25 kg were selected based on their willingness to walk on a motor driven treadmill at 3.2 km/hr with no incline. We have shown previously that gender does not impact cardiovascular responses to activation of the muscle metaboreflex (Laprad et al., 1999). Furthermore, previous studies have shown gender in young adults does not impact sympathetically mediated baroreflex responses elicited via hypotension (Kim et al., 2011; Fu and Ogoh, 2019) as was done per this study. All animals used in this study underwent a minimum fourteen-day laboratory and personnel acclimation period prior to any volitional exercise. All experimental and surgical protocols outlined in this study were approved and comply with the Wayne State University Institutional Animal Care and Use Committee (IUCAC) and National Institutes of Health Guide to the Care and Use of Laboratory Animals respectively.

### Surgical Instrumentation

All anesthetic, analgesic and surgical procedures used in this study have been documented in previous studies (O'Leary, 1993; O'Leary and Augustyniak, 1998; Augustyniak et al., 2000; Hammond et al., 2000; Hammond et al., 2001; O'Leary et al., 2004; Kim et al., 2005a; Kim et al., 2005b; Augustyniak et al., 2006; Sala-Mercado et al., 2007; Ichinose et al., 2008; Coutsos et al., 2010; Kaur et al., 2016; Kaur et al., 2018; Mannozzi et al., 2020; Mannozzi et al., 2021). Briefly, all animals in this study underwent three separate surgical anesthetic events using standardized aseptic surgical techniques with a minimum of a 14-day recovery period between each procedure and prior to any experiments. Approximately 30 min prior to induction of anesthesia for each surgery, animals were sedated with an intramuscular injection of acepromazine (0.2–0.5 mg/kg). Anesthetic induction was accomplished through administration of ketamine (5 mg/kg) and diazepam (0.2–0.3 mg/kg), additionally analgesics carprofen (4.4 mg/kg IV) buprenorphine (0.03 mg/kg IM) and a fentanyl patch (50–125 μg/kg 72 h TD) were administered. Animals were anesthetically maintained pre and intraoperatively with (1%–3%) isoflurane gas. IV cefazolin (30 mg/kg IV) was administered pre and intraoperatively for prevention of acute infection. Post operatively animals were administered acepromazine (0.2–0.3 IV) and buprenorphine (0.01–0.03 IM) as needed. Prophylactic antibiotic cephalexin (30 mg/kg PO) was administered for the duration of the protocol to prevent infection of surgical sites.

In the first surgery a left thoracotomy was performed by entering the 3-4th intercostal space. The pericardium was incised to access the apex of the heart for insertion of a telemetric pressure sensor (Data Sciences International St. Paul, MN) and place 0-Flexon steel pacing leads (Ethicon Summerville, NJ) on the epicardium for measures of ventricular pressure and induction of heart failure respectively. Additionally, the ascending section of the aorta was dissected from its surrounding tissue for placement of a 20 PAU flow probe (Transonic Systems Ithaca, NY, United States) to measure cardiac output. The pericardium and ribs were reapproximated and the chest was closed in layers. All cables and wires were tunneled subcutaneously and exteriorized at the scapulae. Animals recovered for a minimum of 14 days prior to the next procedure.

The second surgery was a left retroperitoneal approach to access the terminal aorta and renal artery. A fluid 19-gauge polyvinyl catheter (Tygon, S54-HL, Norton Murdock Industrial Inc. Akron, OH) was placed into the most cranial lumbar artery branch of the terminal aorta for measures of systemic blood pressure. Additionally, a 10 PAU flow probe (Transonic Systems Ithaca, NY, United States) and two hydraulic vascular occluders (DocXS Biomedical Products Petaluma, CA, United States) were placed around the terminal aorta caudally to the catheter to measure and manipulate hindlimb blood flow for experiments unrelated to the current study. Lastly a 4 PSB flow probe (Transonic Systems Ithaca, NY, United States) was placed around the left renal artery for experiments unrelated to the current study. The retroperitoneal space was closed in layers and all cables, catheters and occluder lines were tunneled and exteriorized at the scapulae. Animals recovered a minimum of 14 days prior to the next surgery.

The final surgical procedure was performed using a single 4–5 cm vertical incision on the neck for placement of a vascular occluder (DocXS Biomedical Products Petaluma, CA, United States) on each carotid artery caudally from the carotid body to be used for baroreceptor unloading. The neck incision was closed in layers and occluder lines were tunneled and exteriorized at the scapulae. Animals recovered for a minimum of 14 days prior to any experiments.

### Data Acquisition and Experimental Procedures

Experiments were performed after a minimum of 14 days post-surgery. For each experiment animals were given 10–20 min to acclimatize to the laboratory environment. After acclimation all the fluid catheter was attached to a pressure transducer (Transpac IV, ICU Medical San Clamente, CA, United States) and the flow probes were connected to their respective flow channels on a TS420 flowmeter (Transonic Systems Ithaca, NY, United States). The telemetric DSI implant for the ventricular catheter was turned on to transmit data to the receiver (Data Sciences International St. Paul, MN, United States). All data was collected and analyzed using Labscribe acquisition software (iWorx Dover, NH). With the animals standing on the treadmill at rest the carotid vascular occluders were inflated for 2 min. The data were averaged over the last 1 minute of carotid occlusion. After a minimum of 20 min recovery at rest or on a separate day, the treadmill was started, and the speed raised to 3.2 km/h and maintained for at least 3–5 min to achieve steady state and the carotid occlusions were repeated. All experiments were repeated in the same animals after induction of heart failure induced via rapid ventricular pacing at 225 beats per minute for 31 ± 5 days. Thus, each animal was observed in every setting and condition: e.g., each animal served as its own control in this longitudinally designed study.

### Data Analysis

Cardiac output, mean arterial pressure, and left ventricular pressure were measured and recorded continuously and all other variables shown in this study were derived from these waveforms or through calculations. Heart rate was derived from the left ventricular pressure wave form. Stroke Volume was determined by Cardiac Output/Heart Rate, Total Vascular Resistance was determined by Mean Arterial Pressure/Cardiac Output. End Systolic Pressure and rates of ventricular contractility were derived from the left ventricular pressure wave. Effective Arterial Elastance, the vascular component of the ventricular-vascular coupling relationship was calculated from two previously described methods. The first method is derived from the ventricle (Kelly et al., 1992a; Chantler et al., 2008; Amin et al., 2011; Chantler and Lakatta, 2012; Mannozzi et al., 2020) (End Systolic Pressure/Stroke Volume) and the second from the systemic vasculature (Sunagawa et al., 1985; Kelly et al., 1992a; Kelly et al., 1992b; Kass, 2005; Borlaug and Kass, 2008; Mannozzi et al., 2020) (Heart Rate x Total Vascular Resistance). Finally Stoke work an index of optimal the ventricular-vascular coupling relationship, was calculated as [(Stroke Volume/1000) x Mean Arterial Pressure]. One-minute steady state averages were taken of each of the measured and derived variables at rest, rest with bilateral carotid occlusion, exercise, and exercise with bilateral carotid occlusion before and after induction of heart failure.

### Statistical Analysis

All hemodynamic data in this study, observed and calculated are reported as means ± standard error. Statistical significance was determined using and α-level of *p* < 0.05. A two-way ANOVA with repeated measures was performed with Systat 13 statistical analysis software. When a significant interaction was observed a C matrix was used to test for Simple Effects to compare individual means. The impact of heart failure on the changes induced by bilateral carotid occlusion in each state rest and exercise was compared using Students Paired T-Tests.

## Results

**FIGURE 1:**
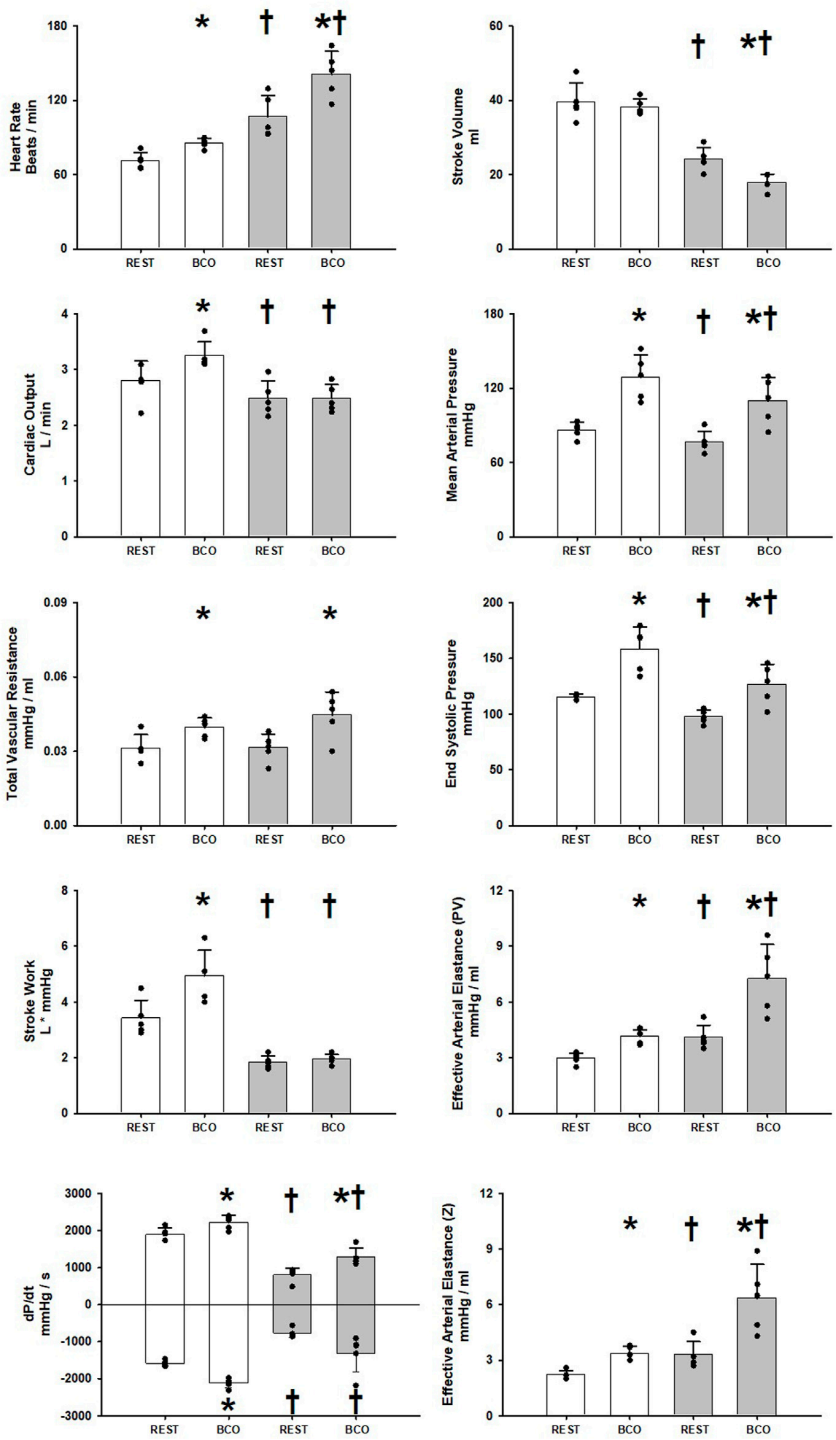
One-minute averaged hemodynamics at rest and at rest with baroreceptor unloading via bilateral carotid occlusion (BCO) before (white bars) and after induction of heart failure (grey bars). Data are reported as mean with errors bars depicting the standard error of the mean. Actual observed data points are overlain on corresponding bar graphs. Statistical significance against the previous exercise workload depicted as *p < 0.05 and significant against previous state at the same workload depicted as ^†^ where *p* < 0.05. (*N* = 5).

Figure 1 shows the one-minute average observed and calculated hemodynamic variables at rest and during rest with baroreceptor unloading via bilateral carotid occlusion (BCO) before and after induction of heart failure. In control BCO caused a significant increase in Heart Rate, Cardiac Output, Mean Arterial Pressure, Total Vascular Conductance, End Systolic Pressure, Stroke Work, dP/dt MAX and MIN and both measures of Effective Arterial Elastance derived from ventricular components (PV) and peripheral vascular components (Z). After induction of heart failure resting Heart Rate, and both measures of Effective Arterial Elastance (PV, Z) were significantly increased. Stroke Volume, Cardiac Output, Mean Arterial Pressure, End Systolic Pressure, Stroke Work, dP/dt MAX and MIN were all significantly reduced at rest after induction of heart failure. In Heart Failure BCO significantly increased Heart Rate, Mean Arterial Pressure, End Systolic Pressure, both indexes of elastance, and dP/dt MAX. Stroke Work, and Cardiac Output did not significantly change in heart failure whereas Stroke Volume significantly fell. Total Vascular Resistance did not significantly change with the induction of heart failure; however, it did significantly increase during BCO at rest in heart failure.

**FIGURE 2:**
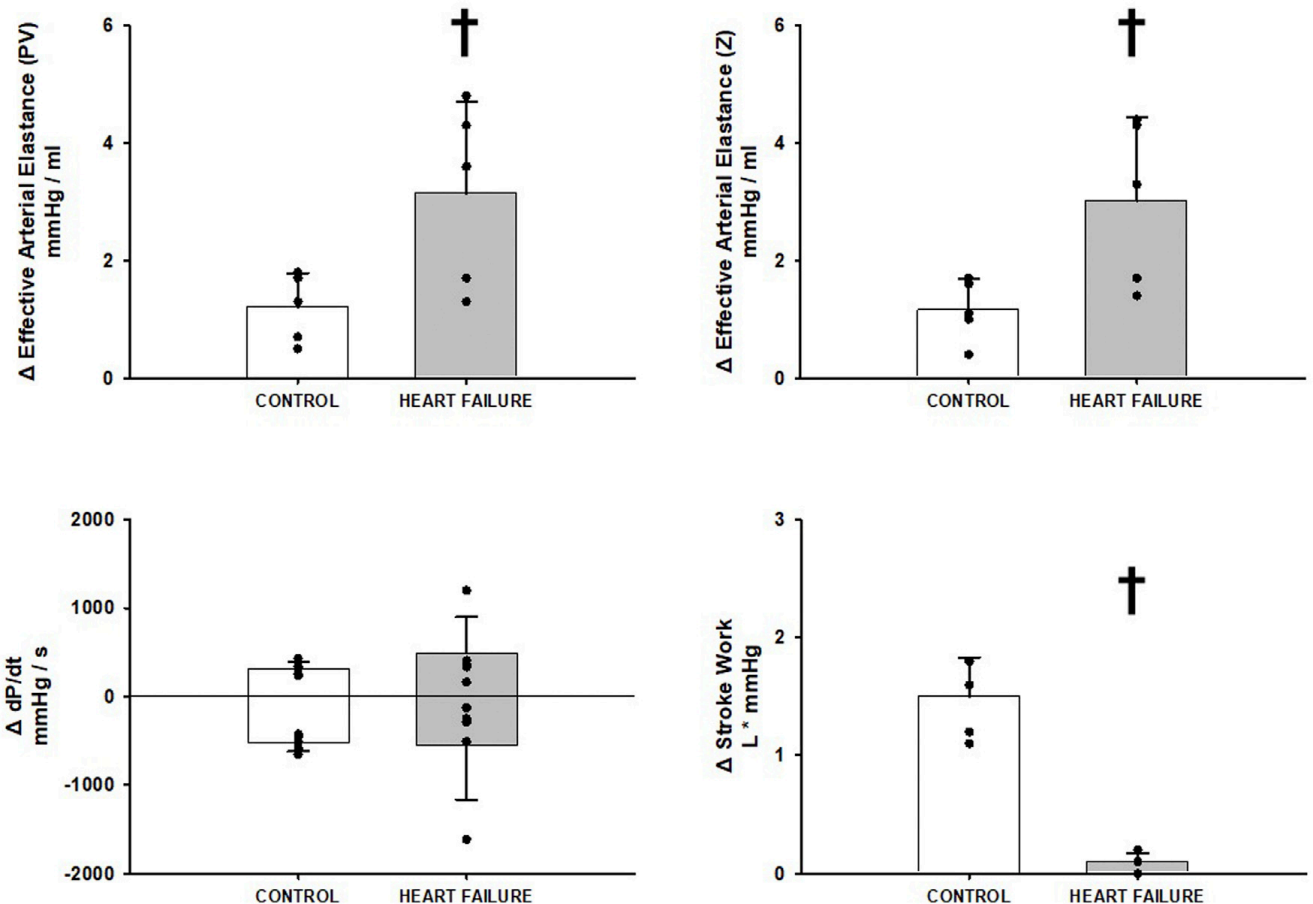
Relative change in hemodynamic variables between rest and rest with baroreceptor unloading via bilateral carotid occlusion in control (white bars) and after induction of heart failure (grey bars). Data reported as means with errors bars depicting the standard error of the mean. Observed data points are overlain on corresponding bar graphs. Statistical significance depicted as ^†^ where *p* < 0.05. (*N* = 5).

Figure 2 shows the changes induced by BCO at rest before and after induction of heart failure in both measures of Effective Arterial Elastance (PV, Z), Stroke Work, and dP/dt MAX and MIN. Heart failure significantly increased both Effective Arterial Elastance (PV, Z) responses to BCO. The Stroke Work response to BCO was significantly attenuated in heart failure and no significant difference was observed in the relative change of dP/dt MAX or MIN during BCO, albeit the baseline levels of both were significantly reduced with heart failure (Figure 1).

**FIGURE 3:**
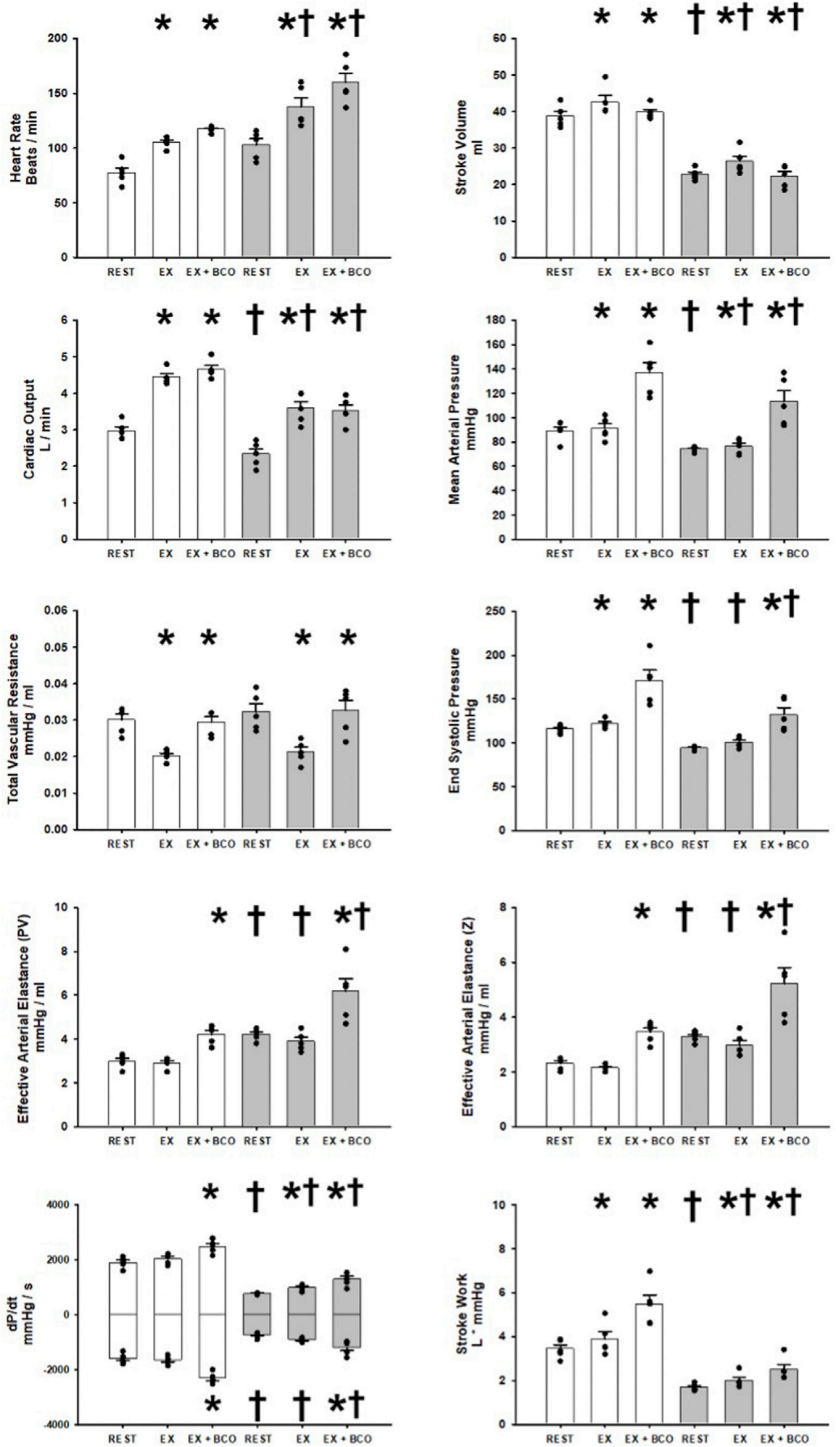
One-minute averaged hemodynamics at rest, exercise, and exercise with baroreceptor unloading via bilateral carotid occlusion (BCO), before (white bars) and after induction of heart failure (grey bars). Data are reported as means with error bars depicting the standard error of the mean. Actual observed data points are overlain on corresponding bar graphs. Statistical significance against the previous exercise workload depicted as *p < 0.05 and significant against previous state at the same workload depicted as ^†^ where *p* < 0.05. (*N* = 5).

Figure 3 shows the averaged observed and calculated hemodynamic responses comparing rest, exercise, and exercise with BCO before and after induction of heart failure. In healthy subjects the transition from rest to exercise induced significant increases in heart rate, stroke volume, cardiac output, end systolic pressure, mean arterial pressure, and stroke work. No significant change was observed in dP/dt MAX and MIN and a significant reduction in total vascular resistance was observed. Activation of the baroreflex during exercise via BCO induced statistically significant increases in heart rate, cardiac output, mean arterial pressure, total vascular resistance, end systolic pressure, both measures of Effective Arterial Elastance (PV, Z), dP/dt MAX and MIN, and stroke work. Whereas stroke volume was significantly reduced with BCO during exercise. After induction of heart failure significant reductions in stroke volume, cardiac output, mean arterial pressure, end systolic pressure, both measures of Effective Arterial Elastance (PV and Z), dP/dt MAX and MIN, and stroke work. Alternatively, no change was observed in total vascular resistance heart rate or dP/dt MIN. In the transition from rest to exercise in heart failure significant increases were observed in heart rate, stroke volume, cardiac output, mean arterial pressure, dP/dt MAX, and stroke work. Conversely significant reductions in total vascular resistance as well as no significant change in either measure of Effective Arterial Elastance or end systolic pressure were observed in the transition from rest to exercise. BCO activation during exercise in heart failure yielded significant increases in heart rate, mean arterial pressure, total vascular resistance, end systolic pressure, Both measures of Effective Arterial Elastance (PV, Z), dP/dt MAX and MIN, and stroke work. In contrast, stroke volume and cardiac output were significantly reduced during exercise with BCO Heart Failure.

**FIGURE 4:**
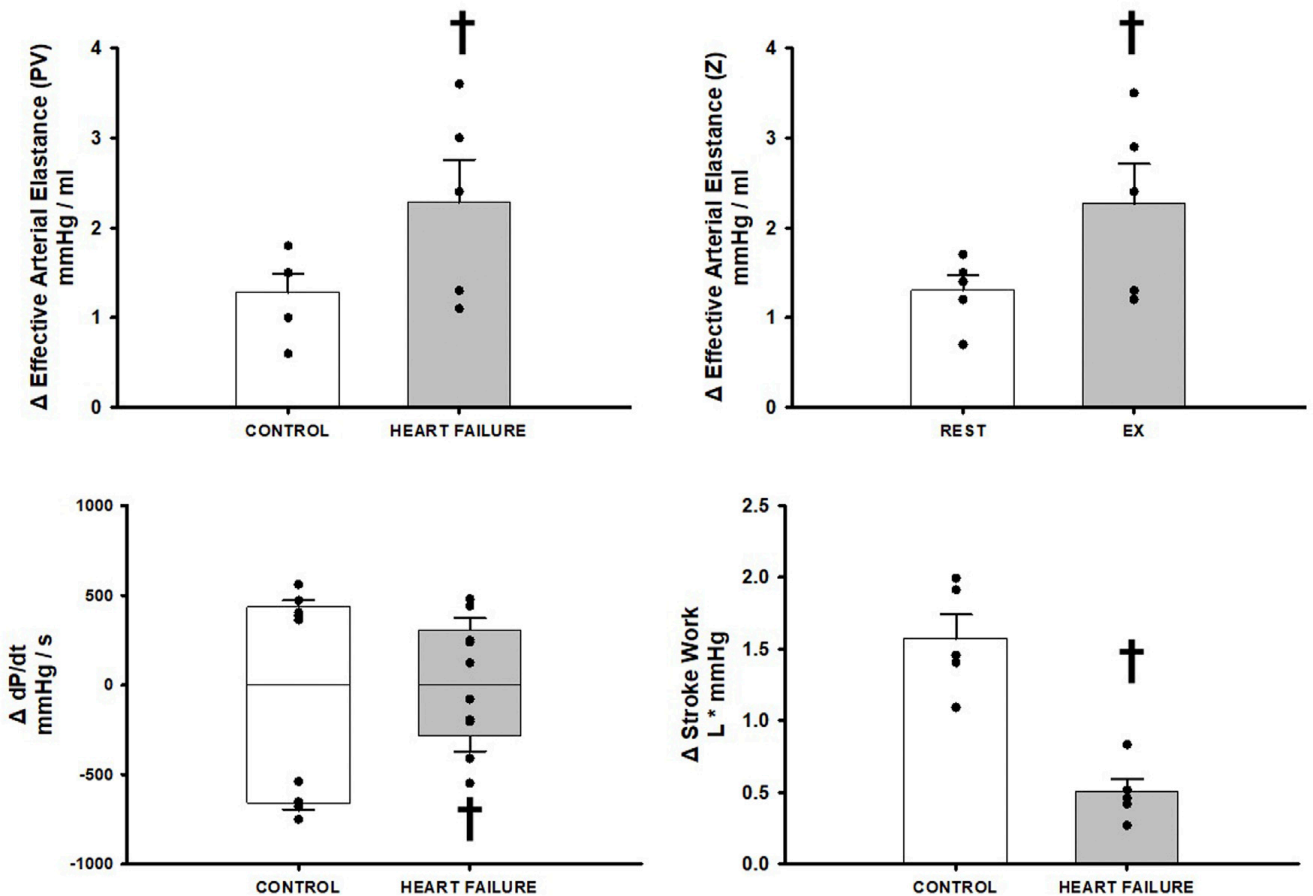
Relative change in hemodynamic variables between exercise and exercise with baroreceptor unloading via bilateral carotid occlusion in control (white bars) and after induction of heart failure (grey bars). Data reported as means with errors bars depicting the standard error of the mean. Observed data points are overlain on corresponding bar graphs. Statistical significance depicted as ^†^ where *p* < 0.05. (*N* = 5).

Figure 4 illustrates the effect of BCO during exercise on indices of Effective Arterial Elastance, dP/dt MAX and MIN, and stroke work, in control and after induction of heart failure. Heart failure significantly increased the reflex increases in both measures of Effective Arterial Elastance and caused a significant reduction in stroke work relative to control. No significant changes were observed on dP/dt MAX however a significant reduction in dP/dt MIN was observed.

## Discussion

This is the first study to demonstrate the impact of baroreflex induced sympatho-activation on the ventricular-vascular coupling relationship in chronically instrumented conscious canines. We used 2 separate indices of Effective Arterial Elastance as one portion of the ventricular-vascular coupling relationship and we simultaneously measured Stroke Work as an index of how optimal the relationship is functioning. We observed that baroreflex unloading via bilateral carotid occlusion in healthy animals at rest and during exercise elicits profound increases in both ventricular and arterial assessments of Effective Arterial Elastance that coincide with significant increases in Stroke work. Thus, in normal animals arterial baroreflex unloading promotes improved ventricular-vascular coupling and thereby likely improves systemic perfusion and arterial blood pressure homeostasis. In heart failure the already uncoupled ventricular-vascular relationship is worsened by arterial baroreflex unloading due to substantial increases in Effective Arterial Elastance that likely contributes importantly to the inability to improve Stroke Work. The inability to improve Stroke Work is a direct index of further significant impairment in ventricular-vascular coupling and thereby a likely reflects an attenuated ability to improve systemic perfusion and maintain blood pressure homoeostasis at rest and during exercise.

In healthy subjects the arterial baroreflex acts as a negative-feedback proportional control mechanism which maintains arterial blood pressure within a normal range on a beat-by-beat basis and can be assessed as spontaneous baroreflex activity or baroreflex sensitivity (gain) (Chen et al., 1991; Iellamo et al., 1997; DiCarlo and Bishop, 2001; Loimaala et al., 2003; Ogoh et al., 2003; Joyner, 2006; Hesse et al., 2007; Drew et al., 2008; Ichinose et al., 2008; Chen et al., 2010; Dutoit et al., 2010; Hart et al., 2010; Sala-Mercado et al., 2014; Hureau et al., 2018). The arterial baroreflex acts to modify blood pressure primarily via modulating autonomic outflow which then induce changes in cardiac output (mostly via changes in heart rate) as well as alterations in total vascular resistance. This dynamic control affords healthy subjects the ability to maintain blood pressure homoeostasis in response to hypotensive bouts engendered by postural changes and rest immediately after severe isometric exercise, through swift retraction of parasympathetic activity and activation of sympathetic outflow. Inasmuch as the arterial baroreflex can combat hypotensive stimuli it can also inhibit hypertensive stimuli such as those elicited by other cardio sympathetic reflexes such as the muscle metaboreflex during exercise (Ichinose et al., 2002; Kim et al., 2005a; Kim et al., 2005b; Iellamo et al., 2007; Drew et al., 2008; Ichinose et al., 2008; Fisher et al., 2010; Ichinose et al., 2012; Choi et al., 2013; Kaur et al., 2016; Ichinose et al., 2017; Kaur et al., 2018). This is a powerful blood pressure raising reflex that occurs during exercise and has previously been observed to maintain optimal ventricular-vascular coupling through large increases in both ventricular maximal elastance as well as Effective Arterial Elastance which together promote robust increases in stroke work (Sala-Mercado et al., 2006; Sala-Mercado et al., 2007; Mannozzi et al., 2020; Mannozzi et al., 2021). However, in healthy subjects the arterial baroreflex buffers the vascular component of the muscle metaboreflex and likely plays a significant role in the maintenance of the ventricular-vascular coupling relationship by modifying sympathetic outflow to the peripheral vasculature (Ansorge et al., 2005; Kim et al., 2005a; Kim et al., 2005b; Iellamo et al., 2007; Ichinose et al., 2008; Kaur et al., 2016; Kaur et al., 2018). In heart failure multiple characteristics of the arterial baroreflex are altered such that they likely contribute to orthostatic and exercise intolerance. However, the exact alterations in the reflex characteristics that likely cause these effects varies depending on the aspect of the baroreflex being assessed. For instance, in heart failure the arterial baroreflex exhibits an attenuated ability to buffer sympathetic outflow during exercise and a decreased response to baroreceptor unloading under pharmacological induced hypotension (White, 1981; Ferguson et al., 1992; Olivier and Stephenson, 1993; Thames et al., 1993; Osterziel et al., 1995; Kim et al., 2004; Kim et al., 2005a; Kim et al., 2005b; Ichinose et al., 2008). Conversely, although buffering capacity is reduced, control of sympathetic activity is maintained in regard to the functional range of baroreflex response (Thames et al., 1993; Wang et al., 1996).

In this study we observed a diminished BCO induced pressor response likely due to the inability to increase cardiac output which was caused by significant reductions in stroke volume. Significant increases in heart rate were observed like as a compensatory mechanism to offset the afterload induced reductions to stroke volume. Additionally, we observed the increase in ventricular performance in healthy animals was significantly diminished in heart failure as observed by reductions in dP/dt MAX and MIN and a reduction in peak increase in end systolic pressure. Thus, the ability to regulate the ventricular component of the ventricular-vascular coupling relationship is likely nearly abolished in heart failure. In addition to these deficits, we observed that in response to BCO both indexes of Effective Arterial Elastance were significantly increased in control and, after the induction of heart failure, the increases in E_a_ were significantly enhanced. By breaking these separate measures of elastance into their components we observe that the vascular component of the ventricular-vascular relationship is impacted by multiple factors. Effective Arterial Elastance PV was significantly increased because of significant albeit attenuated increase in End Systolic Pressure coupled with a significant reduction in Stroke Volume during bilateral carotid occlusion in both exercise and rest. Alternatively Effective Arterial Elastance Z was significantly increased during bilateral carotid occlusion at rest and during exercise because of the maintained increase in Total Vascular Resistance in heart failure coupled with the significantly enhanced heart rate. Together, the ventricular deficits coupled with the enhanced Effective Arterial Elastance significantly impairs energy transfer from the left ventricle and the systemic circulation and thus we observe the significant attenuation of Stroke Work and ultimately these effects are indicative of further ventricular-vascular uncoupling. Furthermore, the enhancements in Effective Arterial Elastance (PV, Z) likely further attenuate not just transfer but the overall propagation of energy throughout the arterial system thereby limiting systemic perfusion and contributing to orthostatic and exercise intolerance.

## Limitations

We used the classic technique of BCO to unload the carotid arterial baroreceptors in order to elicit baroreflex mediated autonomic responses. Previous studies utilizing an isolated carotid sinus technique in conscious dogs have shown that both the arterial pressure and heart rate responses to BCO are similar to those observed in response to reducing pressure in the isolated carotid sinus to sub-threshold levels (Stephenson and Donald, 1980). However, with this approach only one side of the carotid sinus stimulus - response relationship was observed as the responses to maximal activation of carotid sinus baroreceptors could not be performed with the techniques we utilized. This could be a great future interest inasmuch as baseline autonomic activity is changed with heart failure: sympathetic activity is elevated, and parasympathetic activity is lower. In the current study, it is unknown whether further baroreflex mediated increases in sympathetic activity are limited due to the elevated baseline sympathetic tone. However, given that increases in Effective Arterial Elastance, in response to BCO, were significantly greater in heart failure both at rest and during exercise indicates that despite elevated baseline levels, substantial baroreflex—induced increases in sympathetic activity still occurred. With the elevated levels of sympathetic activity in heart failure, it is possible that responses to arterial baroreceptor loading could be affected. These questions await further investigation.

We conclude that arterial baroreflex unloading at rest and during exercise acts to maintain ventricular-vascular coupling and thereby preserve blood pressure homeostasis by improving both ventricular and vascular aspects of the relationship. In heart failure significant ventricular-vascular uncoupling is apparent and drastically impairs any reflex increase in ventricular. Baroreceptor unloading in heart failure induces significant increases in Effective Arterial Elastance that in turn limit ventricular energy transfer as indexed by significant reductions in Stroke Work which likely inhibits propagation of ventricular energy throughout the arterial system. Thus, arterial baroreflex control not only is directly altered by the pathology of heart failure but also baroreflex-induced sympatho-activation actively worsens the ability of the reflex to improve perfusion pressure.

## Data Availability

The raw data supporting the conclusion of this article will be made available by the authors, without undue reservation.
